# Beneficial Effects of Exercise on Depression and Anxiety During the Covid-19 Pandemic: A Narrative Review

**DOI:** 10.3389/fpsyt.2020.587557

**Published:** 2020-11-04

**Authors:** Shaojuan Hu, Lorelei Tucker, Chongyun Wu, Luodan Yang

**Affiliations:** ^1^College of Physical Education and Sports Science, HengYang Normal University, Hengyang, China; ^2^Department of Neuroscience and Regenerative Medicine, Augusta University, Augusta, GA, United States; ^3^Laboratory of Laser Sports Medicine, College of Physical Education and Sports Science, South China Normal University, Guangzhou, China

**Keywords:** physical exercise, depression, anxiety, COVID-19, isolation, quarantine

## Abstract

First reported in Dec 2019, the on-going COVID-19 pandemic has become a public health emergency of international concern (PHEIC). The isolation and quarantine during the COVID-19 pandemic limited the physical and social activities of the population, which contributed to the increased prevalence of mental disorder. Depression and anxiety are the most common mental illnesses conferring a serious impact on individuals' life quality. This review summarizes the mental health consequences of COVID-19, especially for depression and anxiety. Exercise as an intervention for anxiety and depression has been demonstrated in both of the animal studies and human clinical trials. The underlying mechanism including the regulation on the production of brain-derived neurotrophic factor (BDNF), D-β-hydroxybutyrate, synaptic transmission, hypothalamic pituitary adrenal (HPA) axis, tryptophan hydroxylase, GSK3β/β-catenin pathway, neuroinflammation, oxidative stress and PGC-1α1-PPAR axis. In addition, we summarized the exercise strategies to fight against anxiety and depression according to the information from American College of Sports Medicine (ACSM), World Health Organization and recent literatures about physical exercise during COVID-19.

## Introduction

Since the first coronavirus infection (COVID-19) case reported in Dec 2019, the COVID-19 continue to emerge and represent a serious issue to public health (1). In the past several months, increased number of confirmed coronavirus cases and deaths, stay-at-home restriction and tons of information about COVID-19 inevitably conferred impact on people's mental health, especially for people already living with mental disorder (2). According to a perspective article published in the *New England Journal of Medicine*, depression and anxiety may develop to be side effects of COVID-19 after the Covid-19 Pandemic (3). However, the medical care required to large number of COVID-19 cases induced the currently ignorance regarding public mental health during the coronavirus pandemic (1, 3).

According to previous studies, more than 50% of patients with severe acute respiratory syndrome (SARS) and middle east respiratory syndrome (MERS) experienced varying degrees of mental disorder after the outbreak of SARS in 2003 and MERS in 2015 (1, 4), indicating widespread outbreaks of infectious diseases were usually closely related to the increased prevalence of mental disorders (5). Among these disorders, anxiety and depression are the most common mental illnesses conferring a serious impact on individuals' life quality (6). Researchers in China investigated the psychological impact of the COVID-19 during the initial stage of coronavirus and found a high percent of the respondents with moderate to severe depressive (16.5%) and anxiety (28.8%) symptoms (7). Although emergency psychological crisis interventions has been performed to reduce the mental impact induced by COVID-19, changings to the public mental health still exist (8).

The beneficial effect of exercise on improving physical health and fighting disease has be widely studied (9, 10). A large body of evidence suggested regular exercise could significantly reduce the risk of depression, anxiety and considered to be beneficial in the prevention of about 25 conditions (11–14). Furthermore, physical inactivity has been considered as a modifiable risk factor for numerous diseases including depression and anxiety (15). Here, we reviewed the recent studies concerning mental health related to COVID-19 coronavirus and the beneficial role of exercise against anxiety and depression. Exercise type, exercise frequency, exercise intensity and the underlying mechanism will be discussed.

### Method

We conducted a narrative review of recent studies concerning mental health related to COVID-19 coronavirus and the beneficial role of exercise against anxiety and depression.

A literature search within the PubMed and Web of Science databases was conducted. Literatures with clear description of exercise type, exercise frequency, exercise intensity and effect of exercise on anxiety and depression were included.

## Increased Risk of Mental Disorders During The Covid-19 Pandemic

As a “public health emergency of international concern (PHEIC)” for the entire world, COVID-19 has caused a rapid growth in the number of confirmed and suspected cases in the past several months (16). Isolation and quarantine were proven as effective measures contributing to the successful containment of the COVID-19 in China and other regions (17). Isolation was defined as the separation and activity restriction of ill persons with infectious disease to prevent its transmission to others. The definition of quarantine differs from isolation referring to the activity restriction of healthy persons who have been exposed to an infectious disease and may suffer with this disease in the future (18). Although the isolation and quarantine were two of the effective disease-control methods according to previous experience including SARS in 2003 (18), people in quarantine or isolation reported a high prevalence of symptoms of mental disorders (18). For the confirmed and suspected patients, they underwent the fear of the severe consequences and quarantine (19, 20). For the medical service provider, especially those working on the Covid-19 battlefront, they are at both high risk of Covid-19 infection and mental health problems, as they experience the fear and worry about their own health, and the spreading the virus to their relatives and friends (20). For other people, stressors during quarantine, including fears of infection, boredom and frustration, inadequate supplies, inadequate information and financial loss, contributed to poorer mental health (21). In addition, due to the mandatory quarantine procedures in most of the countries suffering the virus, people, especially for people already living with mental disorder, may experience depression, despair and anxiety (2). According to a previous study including 1,210 participants in China during the period January to February, 54% of respondent reported they suffered moderate to severe psychological impacts from the virus. About one-third of them reported moderate to severe anxiety symptoms, and 17% of them reported obvious depressive symptoms (7). The increased level of anxiety and depression in patients and healthy persons were detected after the stay-at-home order in several countries (22–24). As we do not know how long coronavirus disruptions will last, finding an intervention to prevent or alleviate the psychological impacts is urgently needed.

## Exercise as an Intervention for Anxiety and Depression During The COVID-19 Pandemic

The effect of exercise in treating or preventing anxiety and depression has been demonstrated in numerous studies (25–27), and widely accepted as an affordable, non-invasive, and easily accessible measures for individual with mental disorders (28, 29). Recently, a study reported that, as people were rarely able to get access to exercise facilities during the Covid-19 pandemic, exergames based on the combination of exercise with appealing digital games was a potential method to cope with anxiety (30). A 20 min single session exergame at moderate intensity were able to significantly reduce the levels of anxiety in healthy person and 8 week exergames performed 2 days per week (60 min *per session*) was demonstrated to alleviate the anxiety levels in patients (30–32). For Covid-19 patients, relaxation techniques and breathing exercises were recommend as one of the interventions to improve acute anxiety, although more evidence is needed (33). Furthermore, a study on college students demonstrated daily physical activity confers beneficial effects in reducing Covid-19-induced stress and anxiety (34).

## Effects and Mechanisms of Exercise on Depression

Depression is one of the most prevalent mental disease affecting around 340 million people all over the world (35), and exert a significant financial and emotional burden to both families and society (36). Patients with depression presented low levels of mood, feelings of guilt, decreased appetite, poor sleep quality, helplessness, low self-worth, fatigue, psychomotor retardation, low interest in social interaction and sexual activity (37). Stressful events from work place and school or abnormal endocrine function such as hypercortisolism are the common risk factors contributing to depression (38), and about 50% of the depression is determined by gene (37).

Studies on animals demonstrated the beneficial role of exercise on depression depends on the regulation of neurotransmitter, neurogenesis, neurotrophic factors, and cerebral blood flow (Table 1) (51). As reported, exercise could induced the increased level of brain-derived neurotrophic factor (BDNF), which contributed the increased ability against anxiety and depression in mice (52). One of the possible mechanisms was due to the accumulation of an endogenous molecule, D-β-hydroxybutyrate (DBHB), in the hippocampus. The increased DBHB level after long-term exercise could cross the blood brain barriers (BBB) and inhibit class I histone deacetylases, which will specifically improve the expression of BDNF and affect synaptic transmission (52). In a sleep deprivation-induced depression mouse model, exercise was also found to normalize the decreased levels of BDNF, and therefore exert neuroprotective effect and neurotrophic effect (52). In addition, study demonstrated exercise pretreatment could prevent depressive behavior and neurochemical alterations, such as increased levels of norepinephrine (NE), serotonin and its metabolite in the mouse brain, associated with sleep deprivation (42, 44). Furthermore, in a maternal separation-induced depression animal model, the level of 5-hydroxytryptamine (5-HT) and tryptophan hydroxylase (TPH) were decreased in the dorsal raphe. However, treadmill exercise was able to alleviate depression-like behavior through increasing 5-HT and TPH expression (43), and GSK3β/β-catenin pathway were also believed involved in this process (43). In AD patients, depression is a first sign of cognitive decline at the early stages of AD progression (53). In our previous study, long-term exercise training has been demonstrated as an effective approach to prevent depression in transgenic AD rats, involving the important role of neuroinflammation, oxidative stress, 5-HT and its receptor regulated by exercise training (14). In addition, a well-designed study found transgenic mice with overexpression of muscle PGC-1α are resilient to stress-induced depression and control plasma and brain kynurenine/kynurenic acid balance, suggesting PGC-1α1-PPAR axis in skeletal muscle as a novel target of exercise in the prevention of depression (14).

**Table 1:** Summary of the effect of exercise on anxiety and depression in animal studies.

**Study**	**Condition of interest**	**Types of exercise**	**Duration**	**Outcome**	**Molecular/cellular**
Bjornebekk et al. (39)	Depression	Running wheels	5 weeks	Decreased depressive-like behaviors	Increased cell proliferation
Yau et al. (40)	Depression	Running wheels	14 days	Decreased depression-like behaviors	Neurogenesis
Schoenfeld et al. (41)	Anxiety	Running wheels	5 weeks	Reduces anxiety-like behaviors	N/A
Daniele et al. (42)	Depression	Treadmill	1 h for 8 weeks	Prevents depressive behaviors	Reduce neurochemical alterations
Wang et al. (43)	Anxiety	Treadmill	30 min per day for 10 days	Ameliorate effect on anxiety-like behaviors	GSK3β/β-catenin
Gokdemir et al. (44)	Depression and anxiety	Treadmill	6 weeks	Decreased anxious-depressive behaviors	Neurogenesis, serotonin, serotonin 1A receptors
Leem et al. (45)	Depression	Treadmill	50 min per day for 4 weeks	Decreased depressive behaviors	Increased neurogenesis via the activation of Wnt/GSK3β/β-catenin pathway
Agudelo et al. (46)	Depression	Running wheel	8 weeks	Decreased depressive behaviors	PGC-1α1-PPAR axis
Wu et al. (14)	Depression and anxiety	Treadmill	45 min per day; 3 times/week for 8 months	Prevents anxious-depressive behaviors	Improves the levels of 5-HT and its receptor; Decreased neuroinflammation and oxidative stress
Park et al. (47)	Depression	Treadmill exercise	30 min at 5 m/min for 15 days	Decreased depressive behaviors	N/A
Motaghinejad et al. (48)	Depression and anxiety	Treadmill forced exercise	45 min/day at 12–13 m/min, for 5 days/week.	Decreased depressive and anxiety level	N/A
Park et al. (49)	Depression	Treadmill exercise	30 min/day at 2 m/minute for 10 day	Alleviated depressive state	Neuronal activation
Patki et al. (50)	Posttraumatic stress disorder	Treadmill exercise	30 min/day at 10–15 m/min for 2 weeks	Decreased depressive and anxiety level	N/A

Studies on human also provided tons of evidence on the beneficial role of exercise on depression (Table 2) (63–66). In a prospective cohort study, sedentary behavior was positively associated with a higher depression scores at different ages. In contrast, moderate-to-vigorous physical activity were negatively related to symptoms of depression. Therefore, this study suggested that increased light activity and reduced sedentary behavior might contribute to the decreased prevalence of depression (59). Although in another study, there were only small effect in favor of exercise in the inhibition of depression, the small sample size, a high heterogeneity in the participants, interventions and methods of measurement may limit the ability to draw positive results (73). In addition, studies from different countries and regions supported the beneficial of regular exercise on depression. A study including 312 Spanish patients with clinically significant depression over 65 years old reported that at least 60 min daily moderate-intensity regular exercise (muscle strengthening, aerobic exercise, flexibility and balance exercises) could significantly alleviate depression symptoms (60). An 8 year follow-up study in Finland found persons with dyskinesia and a sedentary lifestyle were at higher risk for depression compared with physically active individuals with intact mobility (74). A prospective cohort study from Taiwan reported that three times a week of moderate intensity (at least 15 min per time) continuous exercise could significantly decrease the risk of depressive symptoms, suggesting moderate-intensity regular exercise was beneficial way to improve mental health (61). Furthermore, a study in Myanmar and Vietnam found participant with less sedentary behavior and high physical activity were at lower risk of having depression (62). Depression is also one of the most common complications associated with physical diseases and symptoms (75). For cancer patients, routine care with 40 min per time, three time a week home exercise for 12 weeks significantly reduced the level of depression compared with usual-care group without home-based walking exercise (67). For patients with post-traumatic stress disorder (PTSD), depression is one of the common symptoms (76, 77). Two week fixed bicycle aerobic exercise was able to attenuate PTSD severity and depression symptom (68). According to previous study, depression are very common among the elderly woman, and postmenopausal individuals are vulnerable to depression (78). However, 12 week pedometer-based walking significantly decreased the levels of depression (69). The dysfunction of hypothalamic–pituitary–adrenal (HPA) axis, the increased secretion of corticotropin-releasing hormone (CRH), the impaired responsiveness to glucocorticoids, the increased size and activity of the pituitary were found in patients in depression patients. The ability of exercise on regulating hypothalamo-pituitary adrenal (HPA) axis supported physical exercise may one of the method to improve depression symptoms (79, 80).

**Table 2:** Summary of the effect of exercise on anxiety and depression in human studies.

**Study**	**Condition of interest**	**Types of exercise**	**Amount of exercise**	**Outcome**	**Effect size**
Gong et al. (54)	Prenatal depression	Yoga	12 weeks	Decreased depressive symptoms	Cohen's *d* = 0.59
Maged et al. (55)	Premenstrual Syndrome	Swimming	30 min per time; 3 times/week for 3 months	Decreased depressive and anxiety level	N/A
Wang et al. (56)	Anxiety and stress	Qigong	15–30 min per time; 30 min to 12 weeks	Decreased stress and anxiety level	Cohen's *d* Anxiety: 0.75 Depression: 0.88
Merom et al. (57)	Depression, anxiety, and stress	Home-based walking program	At least 30 min per week	Decreased depressive, anxiety, and stress level	Cohen's *d* Depression:0.36 Anxiety: 0.39
Herring et al. (58)	Anxiety	Resistance or aerobic exercise training	2 weekly sessions	Decreased anxiety symptoms	N/A
Kandola et al. (59)	Depression	Physical activity	Light or moderate-to-vigorous	Attenuated depressive symptoms	Cohen's *d* = 0.904
Lopez-Torres Hidalgo and the DEP-EXERCISE Group (60)	Depression	Aerobic, muscle-strengthening, flexibility, balance-strengthening	At least 30 min; 2 days/week for 6 months	Decreased depressive symptoms	N/A
Chang et al. (61)	Depressive Symptoms	Physical exercise	15 or 30 min per time; 3 or 6 times/week	Decreased depressive symptoms	OR = 0.8, 95% CI 0.66–0.95
Pengpid and Peltzer (62)	Anxiety and Depression	Physical exercise	Low, moderate, and high physical activity	Decreased depressive and anxiety level	N/A
Khanzada et al. (63)	Anxiety and Depression	Regular exercise	N/A	Decreased depressive and anxiety level	N/A
Toups et al. (64)	Major Depression	Walking, jogging, and running	Aerobic exercise	Decreased depressive symptoms	Cohen's *d* 0.539–0.623
Harvey et al. (65)	Depression	Self-reported exercise types	Regular leisure-time exercise	Decreased depressive symptoms	Odds Ratio: 0.98–1.69
Schuch et al. (66)	Depression	Aerobic exercise	Moderate intensity	Decreased depressive symptoms	SMD = 1.33, 95% CI 0.46–2.19, *P* = 0.003)
Chen et al. (67)	Anxiety, Depression	Walking	40 min; 3 times/week for 12 weeks	Decreased depressive and anxiety level	Difference between groups (95% CI) −0.63 (−20.4, 0.78)
Fetzner et al. (68)	Posttraumatic Stress Disorder	Stationary biking aerobic exercise	Six sessions for 2 weeks	Reduced anxiety sensitivity	N/A
Abedi et al. (69)	Depression and anxiety	Pedometer-based walking	4, 8, 12 week intervention	Decreased depressive and anxiety level	8th week (4.2 ± 2.1 vs. 5.4 ± 2.3, *p* = 0.007) 12th week (4.3 ± 2.8 vs. 7.2 ± 2.6, *p* < 0.001)
Meyer et al. (70)	Major Depressive Disorder	Cycle ergometer	30 min of exercise, either (1) at a moderate intensity or (2) at a preferred intensity	Decreased depressive and anxiety level	Cohen's *d* 0.365–0.76
Oliveira et al. (71)	Anxiety and depression	Aerobic exercise	12 week intervention	Decreased depressive and anxiety level	Depression: [4.8(1), *p* = 0.02, b = 1.6]; Anxiety: N/A
Yang et al. (72)	Depression	Exercise intervention	70 min for 60 sessions	Decreased depressive level	N/A

## Effects and Mechanisms of Exercise on Anxiety

According to previous studies, nearly one-half of people diagnosed with depression will also experience comorbid anxiety (81). Anxiety is one of the most common mental health diseases contributing to poor concentration, emotional changes, impaired sleep quality and difficulties in performing daily tasks (82, 83). Due to the typical symptoms, including sweating, shaking, chills, rapid heartbeat, poor mental state and hyperventilation, anxiety was defined as a specific psychiatric disorder (37). As reported by previous studies, 25% of the population reported at least one episode of anxiety disorder during their lifetime, and 6% of men and 13% of women suffer from anxiety disorders in the United States (84). Compared with drug therapy, exercise is considered as an alternative therapy for anxiety disorders, which has lower cost and fewer side effects.

Although compared with human studies, preclinical animal anxiety research was limited by animal models and effective anxiety tests (85, 86), previous studies suggests that exercise can significantly improve anxiety symptoms (14, 26, 37, 87). The possible underlying possible mechanism may rely on the regulation of hypothalamic-pituitary-adrenal (HPA) axis (88), the upregulation of BDNF (52), the improvement of neurogenesis and angiogenesis (89, 90), and the regulation of inflammatory systems (91). HPA axis dysfunction plays an important role in the onset of anxiety (92). The unexpected or/and long-term stress response along HPA axis is able to induce fear, sympathetic disorder and excessive vigilance, which are closely associated with anxiety (88). However, resistance exercise training was able to regulate cortisol levels, which is one of the functional production of the HPA axis (93, 94). In addition to learning and memory, the hippocampus is also an important brain region involved in social cognition and emotion processing (95). According to previous studies, exercise exerted anti-anxiety effects by improving hippocampal neurogenesis and normalizing the neurotransmission of neuropeptide Y (NPY) (96). However, more studies on this mechanism are still needed, as other studies found although exercise can improve adult neurogenesis, the new neurons are not involved in the decreased anxiety-like behavior (41). Similar to depression, the decreased BDNF level is a vulnerability factor for anxiety (97). Numerous studies have found that physical exercise can increase the expression of BDNF in the dentate gyrus (98, 99). Although stress-induced increased was able to down-regulate BDNF levels, interestingly, the physical exercise was found to be able to restore BDNF to pre-stress levels, suggesting that exercise protects against stress-induced decreased level of BDNF (93). The regulation of the inflammatory system by exercise is another possible mechanism against anxiety. As reported in previous study, elevated levels of pro-inflammatory cytokine C-reactive protein (CRP) was associated with anxiety disorders (100, 101). Intriguingly, exercise conferred its beneficial effect on anxiety by regulating inflammatory systems (91, 102).

Similar to depression, studies on human from different countries showed that exercise could attenuate anxiety behaviors. A study from the United States showed that regular physical activity could significantly reduce the risk of anxiety compared to their sedentary counterparts (103). A study including individuals in the Netherlands reported that moderate exercise has a negative association with anxiety symptoms compared with non-exercisers (104). For healthy people, individuals with a single bouts of physical exercise have reported less state anxiety, although they did not investigate the effect of accumulated bouts of exercise on anxiety levels (105–107). However, in other interventional studies, researchers found multiple bouts of exercise for 12 months was associated with significant anxiety reduction compared to control group (108). Additionally, different types of exercise on the anxiety reduction were also reported in numerous studies (109). Yoga, an ancient Eastern practice consisting of breath control, physical postures, and meditation, has shown its beneficial effect on patients with severe anxiety symptoms, although the effect was relatively mild (109). Another study investigating the effect of Tai Chi, a traditional Chinese martial art, on anxiety found that older adults with anxiety receiving medical therapy could benefit from Tai Chi exercise compared with those who only receiving medical therapy (110).

## Exercise Strategies to Fight Against Anxiety and Depression in Covid-19 Pandemic

The beneficial role of physical exercise has been proved in numerous chronic diseases, including heart disease (111), diabetes (112), asthma (113), back pain (114), arthritis (115), cancer (116), and Alzheimer's disease (13). In addition, as mentioned above, tons of evidence has demonstrated the beneficial role of regular physical exercise on the reduction of anxiety and depression (14, 26, 37, 63–66, 87, 117). Although outdoor physical exercise is unavailable during the outbreak of Covid-19, indoor exercise is recommended in view of the positive effect of exercise on boosting immune system (118–120) and alleviating anxiety and depression (14, 26, 37, 63–66, 87, 117).

According to the information from American College of Sports Medicine (ACSM) (121), World Health Organization (122) and recent literatures about physical exercise during COVID-19 (117), the following exercise strategies were summarized: (1) 150 min moderate-intensity or 75 min vigorous-intensity exercise per week, or perform both of them (or modify according to personal or individual specifications). (2) Home-based exercises including knee-to-elbows, plank, back extensions, squats, side knee lifts, “superman,” “Bridge,” chair dips, chest opener, seated meditation, legs up the wall were recommended. (3) For the outdoor activities allowed by the local government, be active in a local park and keep at least 6 feet distance between you and others. (4) The multi-faceted exercise program is recommended, including aerobic, balance, resistance, coordination and activity training are recommended. (5) Do not use public exercise equipment to avoid virus transmission. Notably, these exercise strategies are recommended for healthy individuals in self-quarantine and cannot replace medical guidance.

Although we believe that home-based exercise best avoids viral transmission, a healthy balance between outdoor and indoor physical activities is optimal when possible. The judgement of where to engage in physical activities should be determined according to individual's living environment, economic status, health status, and local restrictions.

## Approaches To Increase Physical Activity Behavior

Although increasing physical activity has demonstrated a beneficial role in fighting anxiety and depression, changing an inactive lifestyle and beginning an exercise regimen is achallenging endeavor for many individuals. According to previous studies, various strategies have been reported as possible approaches to increase physical activity. These include focusing on small quantities of physical activity (e.g., doing exercise during leisure time) (123), improving self-regulation (e.g., learning the benefits of exercise, use of activity trackers to get behavior feedback) (124, 125), strengthening non-conscious processes (e.g., using an enjoyable workout to form an exercise habit) (126), using internet and smartphone apps (127), and increasing accessibility to facilities and environments (e.g., purchasing a treadmill for home use to perform exercise with and compare one's performance with other family members) (125, 128). Also, research suggests a combination of approaches mentioned were better able to lead to improved outcomes (129, 130).

Taken together, the COVID-19 pandemic not only affects physical health, but also mental health (131) Regular physical exercise is for mental health, and able to alleviate the levels of depression and anxiety during COVID-19 pandemic. Staying physically active during the COVID-19 pandemic would contribute to the attenuation of the side effects of COVID-19 on mental health after the pandemic.

## Author Contributions

SH and LY drafted the manuscript. SH and CW prepared the tables. LY and LT edited and revised the manuscript. All authors approved the final version of the manuscript.

## Conflict of Interest

The authors declare that the research was conducted in the absence of any commercial or financial relationships that could be construed as a potential conflict of interest.
